# Immobilization of bacterial spores using eggshells nanoparticles and their effective role in concrete bio-healing process: novel approach

**DOI:** 10.1186/s12896-025-01072-3

**Published:** 2025-11-28

**Authors:** Mai M. Abdelwahed, Amany M. Reyad, Ahmed Abd-Alazim, Gehad Mokhtar

**Affiliations:** 1 Department of Civil Engineering, Faculty of Engineering, Fayoum University, Fayoum, Egypt; 2 Department of Botany, Faculty of Science, Fayoum University, Fayoum, Egypt; 3 Future High Institute of Engineering in Fayoum, Fayoum, Egypt

**Keywords:** Bacterial immobilization, Eggshells nanoparticles, Bio-healing, Concrete strength

## Abstract

**Background:**

Microbially induced calcium carbonate precipitation (MICP) has gained increasing attention as a sustainable bioengineering strategy for improving the strength and bio-healing capacity of concrete. Through biologically mediated CaCO₃ formation, microorganisms can bridge cracks and enhance material cohesion. A persistent challenge, however, lies in maintaining bacterial viability under the mechanically harsh conditions of cementitious systems. To overcome this issue, the present study introduces eggshell-derived nanoparticles (EShN) as an innovative, low-cost carrier for bacterial spores, aiming to improve their protection.

**Methods:**

The size, chemical composition of the EShN, and the spores’ immobilization process were examined using methods such as X-ray diffraction (XRD), scanning electron microscopy (SEM-EDX), and transmission electron microscopy (TEM). The biohealing of concrete and its strengths were investigated.

**Results:**

SEM imaging revealed efficient immobilization of *Bacillus* spores onto the nanoparticle surfaces, while EDX analysis verified calcium carbonate as the main mineral formed within healed cracks. Concrete samples incorporating immobilized bacterial spores exhibited remarkable enhancements in mechanical performance, showing increases of approximately 45, 49.5, and 47.5% in compressive, tensile, and flexural strengths, respectively, compared with negative control.

**Conclusions:**

This research demonstrates that eggshell nanoparticles serve as an effective and sustainable medium for bacterial immobilization, significantly improving both the healing efficiency and mechanical properties of bio-concrete. Overall, this approach provides a promising pathway toward the development of resilient, eco-friendly, and biorepairing construction materials.

## Background

Concrete is one of the most widely used materials in the world due to its low cost, high compressive strength, durability, and outstanding workability [1]. By 2050, global concrete production is expected to increase by up to 23% [2]. The creation of cracks, which affects the efficiency, durability, and protection of concrete structures, is the most significant factor in concrete deterioration [3]. Permeability significantly increases when cracks form a continuous network within the concrete, which negatively impacts its durability by allowing aggressive liquids and gases to penetrate easily [4]. Calcium microbial induced carbonate precipitation (MICP) requires an alkaline pH media, dissolved inorganic carbon, and a calcium resource related to bacteria that operate as nucleation sites through their cell walls [5]. Because the utilization of this large-scale technology is associated with significant financial and environmental costs, one method to reduce these costs and potential environmental liabilities is to use the circular economy to replace traditional carbon and calcium sources with garbage [6].

Bio-healing concrete based on microorganisms that can autonomously repair cracks without the need for human intervention. Recent studies have explored a variety of microbial techniques and materials to enhance the bio-healing efficiency of such systems [7, 8]. Within the microbial cell, on its surface, or even further away inside the concrete, the bacterial conversion process can take place. By adding bio-mineralogy material to concrete, a new substance known as “bacterial concrete” was produced. It explains a novel concrete type that uses microbiologically induced CaCO_3_ precipitation for selective cementation in order to repair microcracks [5].

Bacteria can be placed directly on concrete, where they will remain viable for up to four months, or they can be immobilized by encapsulating and absorbing the bacteria for increased vitality [9, 10]. The carrier units increase the bacteria’s persistence in concrete by preventing them from being stressed during mixing and hydration reactions. The adherence method is recommended for immobilizing bacteria due to its low price [11].

Bacteria must meet certain criteria in order to be used as a curing agent in concrete concrete; for example, 1: Their ability to produce calcium carbonate in concrete despite an alkaline environment. 2: It must contain a significant quantity of calcium carbonate and be resistant to the effects of calcium ion concentration even at high pressure. 3: Using a lot of oxygen and reducing steel corrosion should be oxygen-brilliant. Bacteria serve as a catalyst, which is the primary process by which bacterial cracks heal, converting the components of the precursors into an efficient filler material [12]. It should be mentioned that throughout the mixing and cement hydration processes, there’s a chance that the bacterial cells or spores could get damaged [13]. Furthermore, damaging the microorganisms during mixing due to mechanical forces may occur; consequently, it is advisable to immobilize bacteria or spores before adding them.

Nanomaterials exhibit new properties, including microscopic size effects, surface effects, filling the internal pores making the paste denser, and adsorption properties that macroscopic materials lack [14]. The use of nanomaterials can regulate the hydration process of cement, which in turn improves the mechanical properties and durability of the hardened paste [15]. Because of their characteristics, such as surface interactions and increased surface area, these materials could be able to successfully protect microorganisms in the cement matrix [16]. However, nanoparticles have cost limitations [15]. Uniform dispersion of the nanomaterials in the mixture, which necessitates the employment of non-standard equipment (such as a sonicator), is the largest application difficulty in in-situ settings [16].

Khaliq and Ehsan [17] examined the capability of concrete for self-healing using bacteria (*B. subtilis*) that were immobilized on graphite nanoparticles (GNP). Results demonstrated that the specimens had pre-cracked at an early stage. GNP guaranteed optimal crack-healing effectiveness with up to 810 μm of healed crack. Pre-cracked samples at 14 and 28 days exhibited improved results from bacteria encased in the lightweight aggregate. According to Khushnood et al. [13], immobilized *B. subtilis* bacteria on graphite platelets allowed cracks as deep as 1300 μm to heal in addition to strengthening cement mortars. Thus, the unique benefit of using nanoparticles as bacterial carriers is that the MICP process can fill large cracks up to 1710 μm. Because of their characteristics (such as surface interactions and improved surface area), these materials, despite their nano size, have the ability to successfully preserve microorganisms in the cement matrix area.

The biggest problem when using nanomaterials in in situ environments is making sure they are evenly spread out in the mixture, which requires special tools. Expanded perlite’s high porosity and high water absorption made it an effective carrier material for healing chemicals. When cracks form in the concrete matrix, the high porosity structure can give the immobilized bacteria in the concrete enough oxygen, and the high water absorption can expose the bacteria to enough water [18–20].

In a study by Zhang et al. [18], *B. cohnii* was immobilized using expanded perlite (EP) and expanded clay (EC), two porous carrier materials. The efficacy of the cracks’ healing was compared to samples that directly included the control and identical bacteria without the use of a healing agent. Cracks with a width of 0.45 mm and 0.79 mm, respectively, healed after the initial damage; samples containing EC and EP were left to heal for 28 days. The direct addition of bacteria needed 0.39 mm, while the control sample value was just 0.25 mm. Jiang et al. [21], who coated impregnated EP with various protective layers, reported that the diameter of the restored cracks varied, ranging between 0.39 and 1.24 mm for different outer layers. The particles When not covered in any layer, the maximum repair width was less than 0.27 mm. Wiktor and Jonkers [22] demonstrated that enlarged clay particles containing calcium lactate and bacteria were an effective means of enhancing concrete’s ability for self-healing. They showed an increase in the fully mended fracture from 0.18 mm in the control sample to 0.46 mm in the sample treated with the healing agent. Several investigations have found that the inclusion of ceramsite, expanded clay, or lightweight aggregates have not significantly changed the properties of mortars and concretes [23–25].

Thus, this study used eggshells nanoparticles (EShN) as bacterial immobilization carrier units. However, the immobilization of bacteria in bio-healing concrete has previously been studied using different materials. The use of materials produced from biowaste, such as eggshells in nanoparticulate form, is still relatively unexplored in this field. Calcium carbonate, the main component of eggshells, not only offers bacterial spores a suitable matrix but also helps to improve the concrete mechanical characteristics. The immobilization of bacterial spores on eggshells-derived nanoparticles for concrete bio-healing has not been previously documented in the literature. The conversion of eggshells into nanoparticles greatly increases their surface area, porosity, and bio-affinity, which improves spore attachment, protection, and viability under the harsh alkaline environment of concrete, thus representing a novel and sustainable approach.

## Methods

### Preparation of eggshells nanoparticles (EShN)

The eggshells wastes were provided from poultry farms located at EL Azab, Fayoum, Egypt. After being cleaned with tap water, the eggshells waste was left to dry for a few days in the sun. The eggshells were crushed, ground into a powder, and then passed through 100 μm sieves. Eggshells units were dried in an oven at a temperature around 100–110°C for 24 h to remove any remaining moisture. After drying, eggshells nanoparticles (EShN) were achieved using a ball mill for around 90 min, followed by a disc mill for an additional 10 min [26, 27]. SEM-EDX analysis was obtained using a Carl Zeiss Sigma 500 VP Jeol JSM–6390 apparatus [28]. The transmission electron microscopy (TEM) of the nano eggshells sample was done using a JEOL-2100 electron microscope that works at 200 kV. TEM is an analytical technique was used to visualize the smallest structures in matter, and it can reveal stunning detail at the atomic scale by magnifying nanometer structures up to 50 million times [29]. The X-ray diffraction (XRD) pattern of the nano eggshells sample was recorded using a Bruker A8 Advance diffractometer operated at 40 mA and 40 kV. The diffraction data were collected over a 2θ range of approximately 5° to 80°, with the intensity measured in arbitrary units (a.u.) [30].

### Bacterial strain and immobilization of spores

In this study, a bacterium, *Bacillus paramycoides* strain G10 (GenBank accession number: MZ430955) [28], was used. Spores of the bacterial strain were obtained by culturing the bacteria in an alkaline nutrient broth for 72 h. Multiple centrifugation processes using dual-sterilized tap water were performed to capture the bacterial spores. For 35 min, suspensions were heated at 80°C to deactivate any vegetative cells. The usual count cultivation-dilution technique quantified the viable spores’ number in suspensions [31]. EShN was used to immobilize the bacterial spores using the force of electrostatic attraction. A sonicator operating at 500 W for two minutes was used to disperse the EShN (125 g/L) in distilled water. After that, the bacterial spores were added and shaken in an incubator at 30°C and 150 rpm for 30 min.

### Experimental design

Table 1 shows mortar and concrete mixture design. Three mortar mixtures were compared in this research. Mixture (1): the negative control, there are no spores present in the combination (designated as MC). In mixture (2), the positive control mortar mixture contains eggshells nanoparticles as a replacement for cement ingredients (designated as MN). In mixture (3), 1.3 × 10^7^ spores/cm^3^ of bacterial spores were immobilized on eggshells nanoparticles as bacterial carriers (designated as MNB). All mortar mixtures, beams (40 × 40 × 160 mm) were used and tested on the 28th day after casting and they were loaded until cracked. The resulting cracks have been observed.

**Fig. 1:**
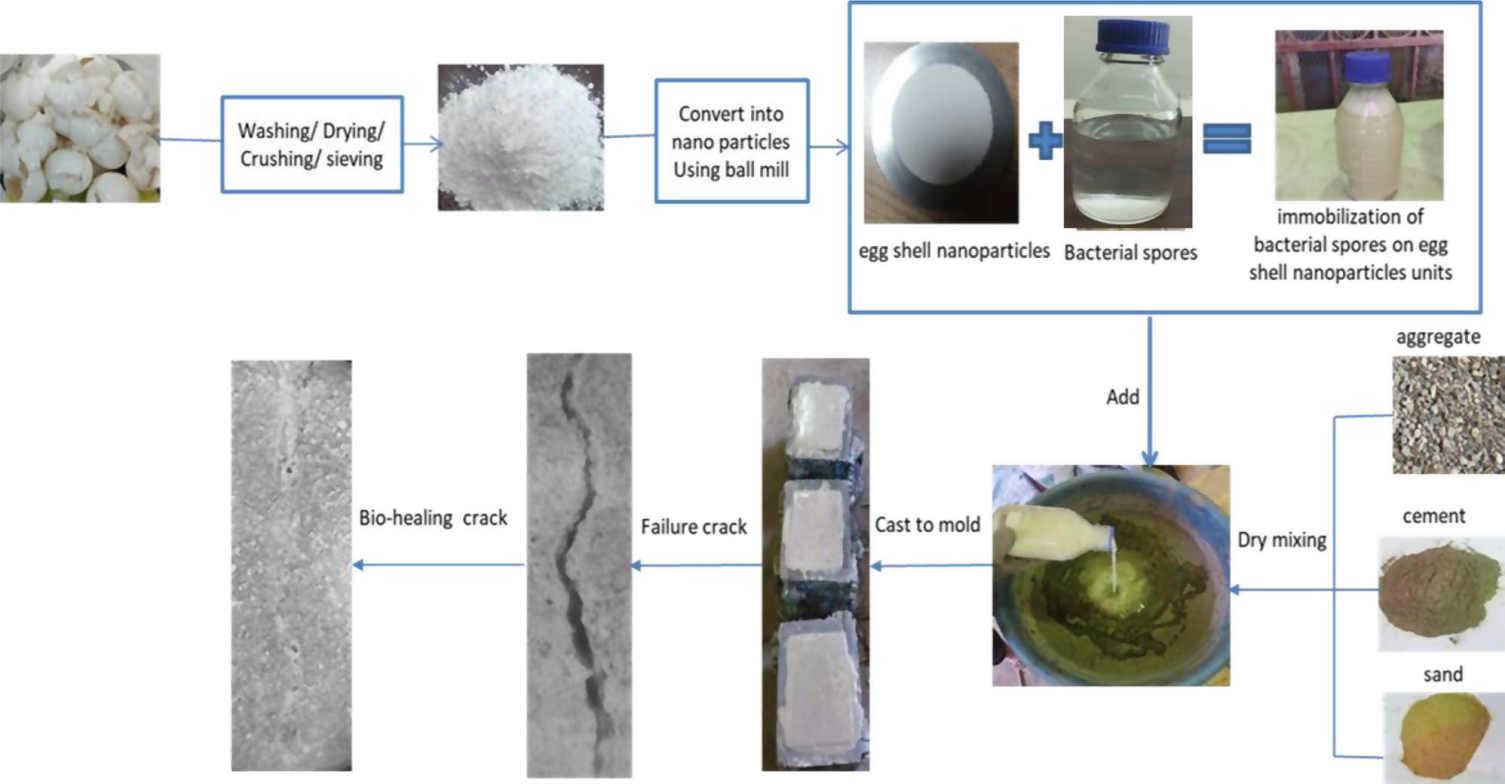
Schematic diagram for the preparation of eggshells nanoparticles and bio-healing mixture

The compressive, flexural, and indirect splitting tensile strengths were tested for hardened concrete. 150 × 150 mm cubic, 100 × 100 × 500 mm beam, and 150 × 300 mm cylinder were used for three concrete mixtures. Mixture (4), the negative control, there are no spores present in the combination (designated as C). In mixture (5), the positive control mortar mixture contains eggshells nanoparticles as a replacement of the cement ingredient (designated as N). In mixture (6), 1.3 × 10^7^ spores/cm^3^ of bacterial spores were immobilized on eggshells nanoparticles as bacterial carriers (designated as NB). The mixtures were designed, and a testing program was conducted in accordance with ECP [32]. For the mortar and concrete mixtures, two grams of yeast extract, 65 g of urea, and 40 g of anhydrous calcium chloride were mixed in sterilized distilled water to make the bio-based mixtures. After that, the mixture was agitated for two minutes; 17.5 kg/m^3^ for each eggshells nanoparticle and bacterial spore solution were added. Figure 1 shows a schematic diagram for the preparation of EShN and bio-healing mixture.

All specimens were taken out of the molds and cured in water after a 24-hour casting period. The compressive, flexural, and indirect tensile strength tests were carried out for hardened concrete after 28 days. Bio-healing was observed and the growth of bacterial spores and detection of calcite were done using SEM-EDX analysis.

**Table 1:** Mortar and concrete mix design

Mixture	Water (kg/m³)	Cement (kg/m³)	Fine aggregate (kg/m³)	Coarse aggregate (Kg/m^3^)	Eggshells nanoparticles weight (kg/m³)	Bacterial spores	Type of mixtures
MC	140	350	1050	-	0	No bacterial spores	Mortar mixtures
MN	140	332.5	1050	-	17.5	No bacterial spores	
MNB	140	332.5	1050	-	17.5	Bacterial spores	
C	140	350	675.5	1340.2	0	No bacterial spores	Concrete mixtures
N	140	332.5	675.5	1340.2	17.5	No bacterial spores	
NB	140	332.5	675.5	1340.2	17.5	Bacterial spores	

MC: Refer to the mortar mixture without nanoparticles and without bacterial spores

MN: Refer to the mortar mixture with nanoparticles and without bacterial spores

MNB: Refer to the mortar mixture with bacterial spores immobilized on nanoparticles

C: Refer to the concrete mixture without nanoparticles and without bacterial spores

N: Refer to the concrete mixture with nanoparticles and without bacterial spores

NB: Refer to the concrete mixture with bacterial spores immobilized on nanoparticles

### Statistical analysis

SPSS Statistical Package Program version 23 was used to statistically analyze the data using a one-way analysis of variance (ANOVA) test. The treatments’ means were analyzed. When the differences were substantial, using the Duncan multiple range test. All tests had a significance level of *P* ≤ 0.05. The means ± standard error (SE) are used to express the results.

## Results and discussion

### Nanoparticle characterization

**Fig. 2:**
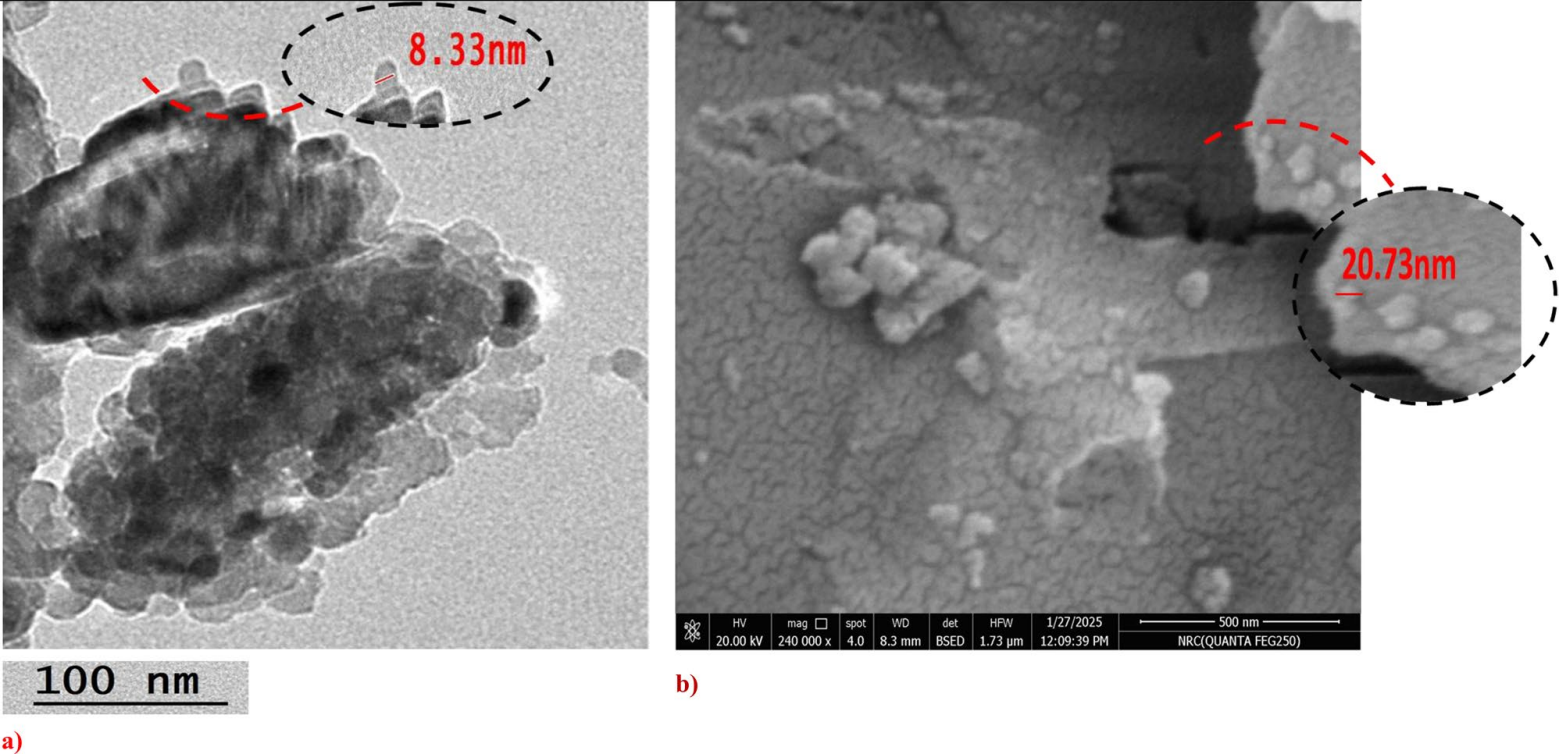
**a**) TEM and **b**) SEM images show the nanoscale size of the eggshells powder units

**Fig. 3:**
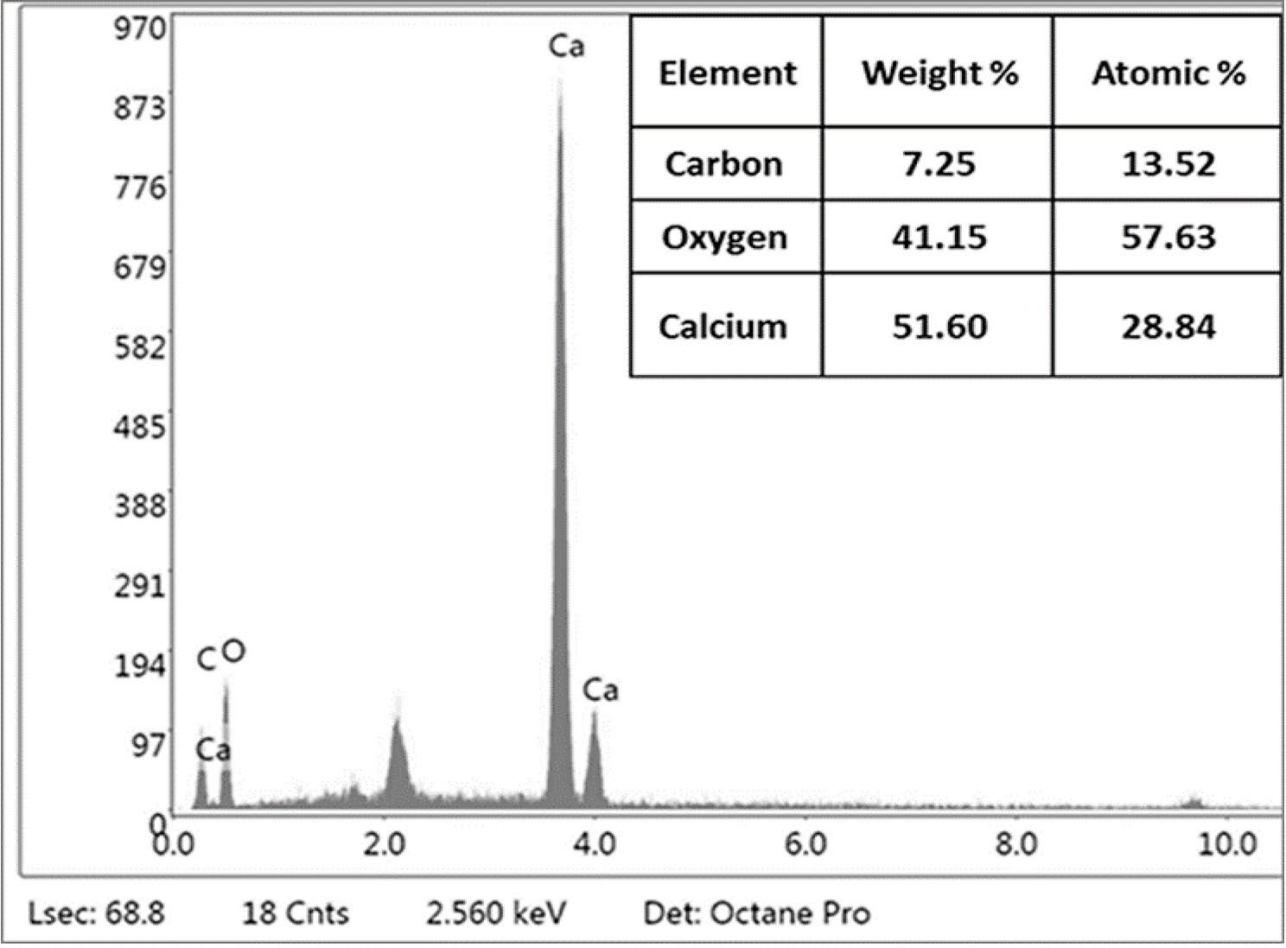
EDX analysis shows chemical composition of eggshells nanoparticles (CaCO_3_ like structure)

**Fig. 4:**
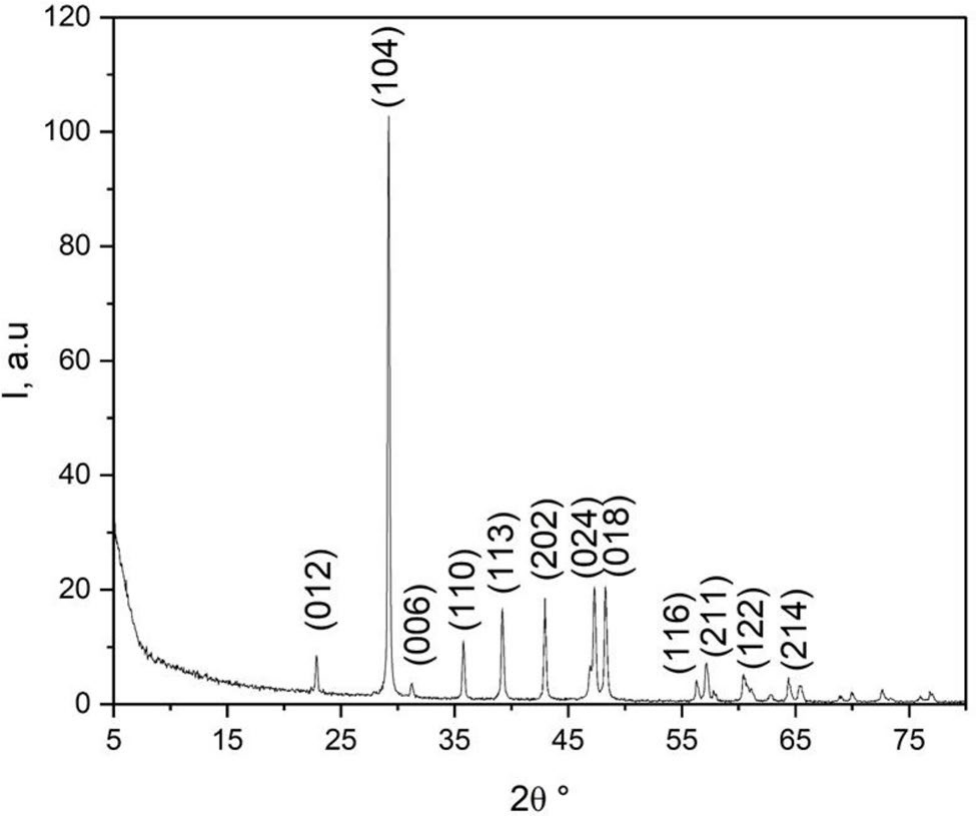
The XRD analysis confirms that the sample is highly matched with crystalline rhombohedral CaCO_3_ (calcite) without any detectable impurities. This confirms the EDX analysis

Techniques like transmission electron microscopy (TEM), scanning electron microscopy (SEM-EDX), and X-ray diffraction (XRD) were used to check the size and chemical makeup of the EShN, as shown in Figs. 2, 3 and 4. The results show that the nanoparticles are very small, but there are some groups of nano-units visible in the images. Using EDX to reveal the chemical composition and XRD to confirm it, it was found that the sample is highly matched with crystalline rhombohedral CaCO₃ (calcite) and contains no detectable impurities; this confirms the EDX analysis. Crystallographic planes and peak analysis show distinct peaks at distinctive diffraction angles that correspond to particular calcite crystallographic planes, including (012), (006), (110), (113), (202), (024), (018), (116), (211), (122), and (214). The most intense peak is observed at 2θ ≈ 29.5°, corresponding to the (104) plane, which is a signature reflection of rhombohedral calcite [33]. The indexed peaks and their relative intensities align well with standard data for calcite, affirming phase purity. No additional peaks related to aragonite or vaterite were detected, indicating the absence of phase impurities [33].

### Bacterial spores’ immobilization

**Fig. 5:**
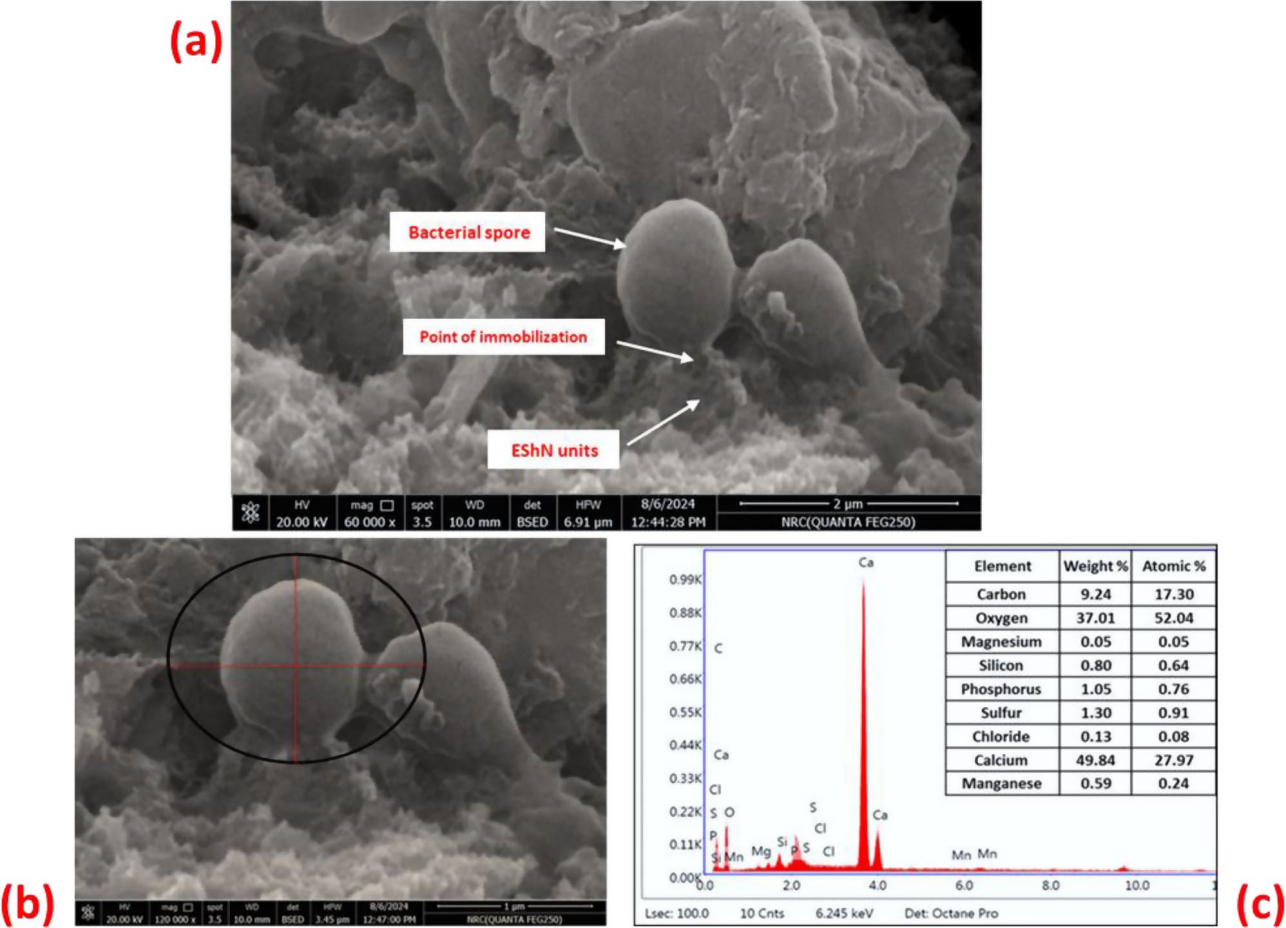
**a**) The SEM micro-image shows the immobilization position of bacterial spores on eggshells nanoparticles units. **b**) Spores attached with eggshells Nanoparticles units (see the black circle). **c**) EDX analysis for bacterial spores confirms their chemical composition

Figure 5 shows the immobilization of bacterial spores on EShN. Spores were attracted by the number of EShN units in a position called the attachment point (see Fig. 5). A sonicator operating at 500 W for two minutes was used to disperse the EShN (125 g/L) in distilled water. After that, the bacterial spores were added and shaken in an incubator at 30°C and 150 rpm for 30 min. The SEM-EDX analysis for the bacterial spores attached to eggshells nanoparticles confirms the chemical composition of spores immobilized on EShN (see EDX analysis in Fig. 5).

### Bio-healing screening

#### Bio-healing of mortar cracks

**Fig. 6:**
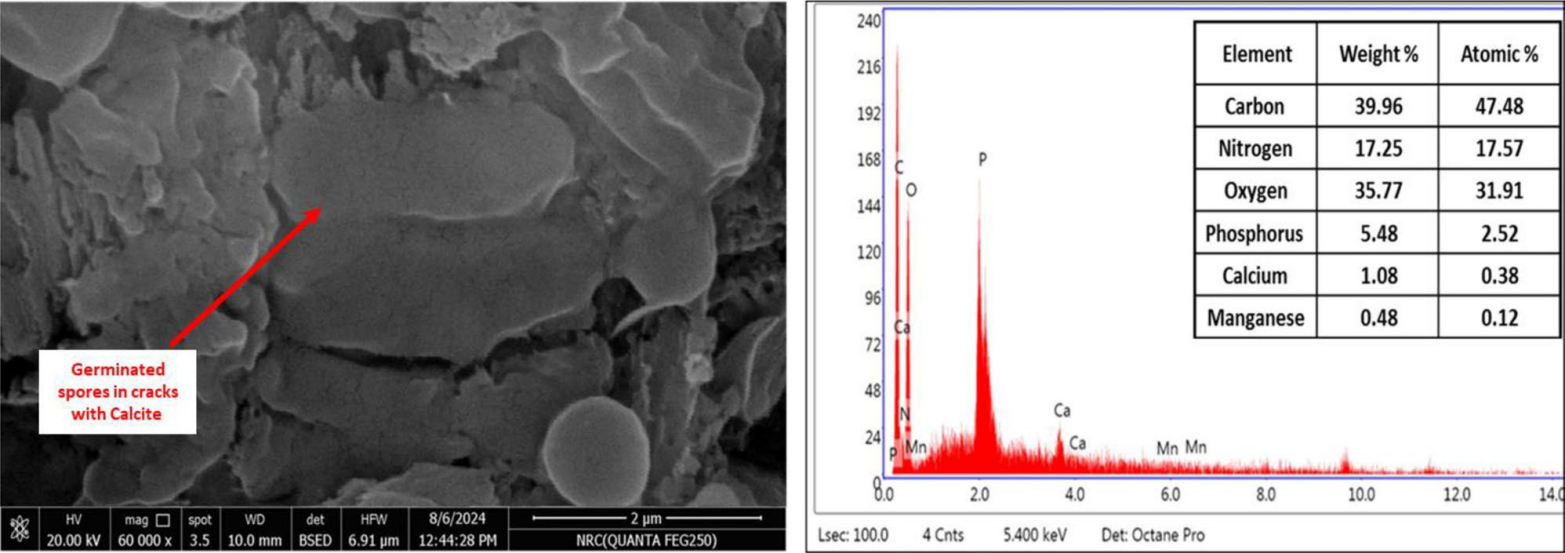
SEM- EDX analysis shows crack healing after bacterial spores’ germination and calcium carbonate precipitation

Completely healed specimens were observed after 28 days by immobilized bacterial spores on EShN. SEM-EDX analysis shows crack healing after bacterial spores’ germination and calcium carbonate precipitation Fig. 6. The improved precipitation rate of calcium carbonate particles may be the consequence of the specimens containing EShN absorbing more bacteria. Calcium carbonate that was found in nanomaterials aided in giving concrete a consistent structure. Consequently, the addition of bacteria and nanomaterials to the mixture results in a more regular crystal structure in concrete. Additional calcium oxide (CaO) is present in concrete that contains waste eggshells particles, which is necessary to create secondary C-S-H gel. As a result, waste eggshells particles may improve the microstructure of concrete by lowering the void volume and partially replacing cement without changing the water absorption value [34].

#### Bio-healing of concrete cracks

**Fig. 7:**
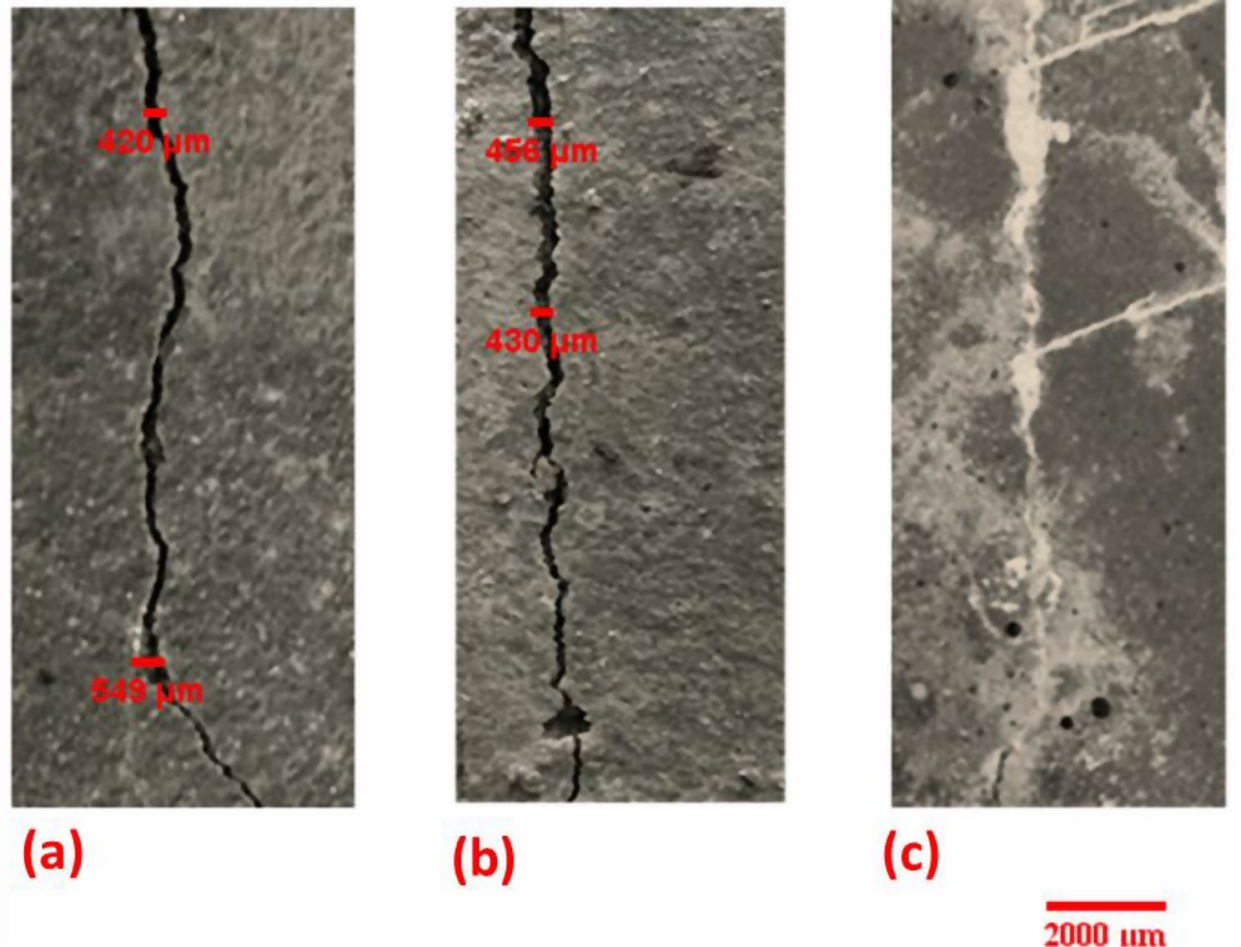
Photo images (10x) show the cracks of concrete specimens healing after 28 days pre-cracking. **a**) Negative control (without nano units or bacterial spores, **b**) Positive control (with nano units and without bacterial spores, **c**) With bacterial spores immobilized on nanoparticles

The photo images in Fig. 7 show the crack healing of concrete specimens after 28 days of water curing of previously cracked specimens. It was observed that the samples with bacterial spore incorporation showed positive restorative results compared to the negative control sample. Following 28 days, immobilized bacterial spores on EShN showed fully healed specimens.

Micrographs from the SEM that demonstrated the reaction product morphology confirmed the obtained results that bacterial integration specimens result in better bio-healing efficiency than control. This is comparable to what was observed by the cement that was hydrated [35–38]. Our findings demonstrated the deposition of CaCO_3_ on the surfaces of the microorganism cells and within the mortar pores in the immobilized bacterial spores’ integration technique. When the bacteria were immobilized on eggshells nanoparticles, which protected them from harsh conditions, they showed the best healing. As cracks appeared, the embedded bacterial spores activated, and their metabolism provided the necessary nutrients that generated CaCO_3_ [39]. The internal structure of concrete is made more compact *via* the constant production of calcium carbonate by bacteria, urea, and calcium chloride supplied as chemical precursors [40]. Fujita et al. [41] demonstrated how particular calcite crystals were screened out of the bio-based concrete and how the untreated specimens’ matrix appeared amorphous with no visible crystal development. The unique benefit of using nanoparticles as bacterial carriers is the large gaps that the MICP method can cover [16]. Nanoparticles are highly reactive due to the vast surface area and small size. Despite their nano size, they can improve the characteristics of concrete and effectively preserve microorganisms within the cement surface area [16].

### Compressive strength

**Fig. 8:**
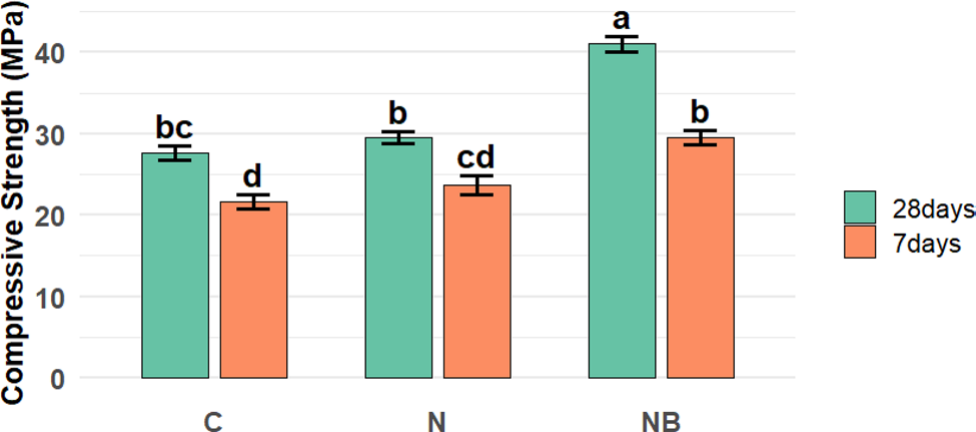
Presents the compressive strength (Mean ± SE) of the concrete specimens at 7 and 28 days of water curing, along with the 95% confidence intervals (CI). At both curing time, the NB specimens showed the highest compressive strength (29.57 ± 0.87 MPa at 7 days and 41.00 ± 1.00 MPa at 28 days), followed by N (23.67 ± 1.20 MPa at 7 days and 29.50 ± 0.76 MPa at 28 days), whereas C exhibited the lowest values (21.67 ± 0.88 MPa at 7 days and 27.67 ± 0.88 MPa at 28 days). A two-way ANOVA revealed that both mixture type and curing time had significant effects on compressive strength (F = 101.509, p < 0.001, ges = 0.894 for time; F = 72.038, p < 0.001, ges = 0.923 for mixture type). Moreover, a significant interaction effect was found between mixture type and time (F = 5.716, p < 0.05, ges = 0.488), indicating that the influence of nanoparticles and bacterial spores on compressive strength varied depending on the curing period. *(GES): (Generalized Eta-Squared is a measure of effect size used in ANOVA to indicate the proportion of variance explained by a factor). C: Refer to the concrete mixture without nanoparticles or bacterial spores. N: Refer to the concrete mixture with nanoparticles and without bacterial spores. NB: Refer to the concrete mixture with bacterial spores immobilized on nanoparticles

The experimental results showed that the immobilized bacterial spores positively influence the compressive strength of the concrete, as presented in Fig. 8. The concrete specimens supplemented with immobilized bacterial spores increased the compressive strength after 7 & 28 days by 34 & 45%, respectively, as they were compared to the negative control samples, and 26.4 & 33.4%, respectively, as they were compared to the positive control (with eggshells nanoparticles as a replacement for cement). Our results are corroborated by Jhatial et al. [42] who found that when 10% eggshells powder was substituted for cement. The compressive strength improved by 16.9%. When 15% eggshells powder was substituted for cement, and the compressive strength increased by 26.6%.

Microbially induced calcium carbonate precipitation (MICP) is a natural process that uses bacteria to help create calcium carbonate (CaCO_3_) in concrete. This process has been used to improve the mechanical properties of concrete by forming calcium carbonate bonds with the concrete matrix. The procedure can help to improve the overall bonding strength between the aggregate and the cementitious phase, resulting in an increase in compressive strength [43, 44]. MICP, which fills micro-cracks and improves mechanical characteristics, is responsible for an increase in compressive strength. The combination comprising EShN has a high early compressive strength due to the highly specific surface areas [13]. There are three possible explanations for the concrete specimens’ increased compressive strength. First, the specimen hardened more quickly due to its stronger surface area and strong electrostatic attraction. Strength was increased by combining eggshells nanoparticles with bacteria, and the combined effect of both led to the closure of cracks. Secondly, the eggshells nanoparticles then functioned as filler product and helped create a tighter microstructure because of their ultrafine size. Furthermore, the immobilizing medium with nanoscale size was effective for the uniform dispersion of microorganisms in the matrix, effectively sealing nano/micro cracks [45–47]. Finally, EShN increased the durability of concrete since it contains a lot of calcium oxide, which is necessary for hydration in concrete production. Additionally, it reduces water absorption as a result; this concrete might be very resistant to environmental stresses such as those caused by acids and sulfates [48]. EShN increases the compressive strength of concrete throughout all age ranges. During hydration, CaCO_3_ joins forces to form an enhanced hydration process [49]. Hussein et al. [50] demonstrated that nano-eggshells powder was utilized to increase the cement mortar’s compressive strength. At a later age, they discovered that CaCO_3_ enhances the mechanical properties of cement mortar.

### Tensile strength

**Fig. 9:**
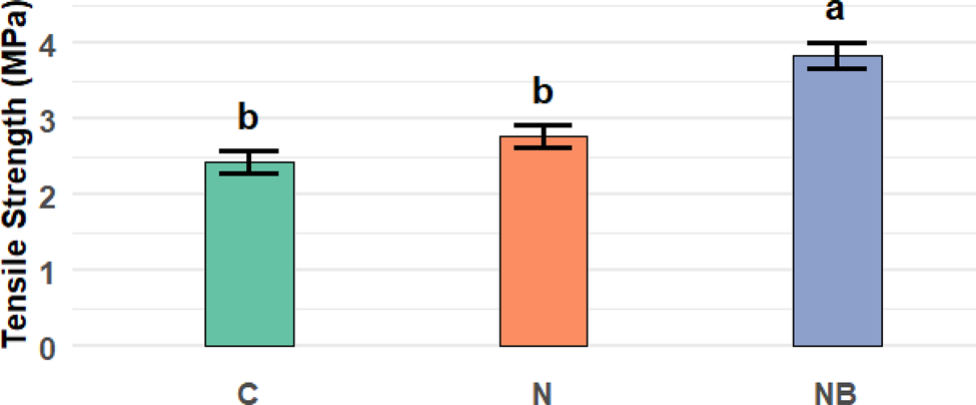
Shows the mean values of the tensile strength (MPa) of the concrete specimens after 28 days of water curing, expressed as Mean ± SE along with the corresponding 95% confidence intervals (CI). Among the tested groups, NB specimens exhibited the highest tensile strength (3.83 ± 0.18 MPa), followed by N (2.77 ± 0.15 MPa), while C showed the lowest value (2.43 ± 0.13 MPa). A one-way ANOVA revealed a statistically significant effect of the mixture type on tensile strength (F= 21.879, *p* = 0.002, ges = 0.879), indicating that the incorporation of nanoparticles and bacterial spores contributed to improving the tensile strength of the concrete specimens. C: Refer to the concrete mixture without nanoparticles or bacterial spores. N: Refer to the concrete mixture with nanoparticles and without bacterial spores. NB: Refer to the concrete mixture with bacterial spores immobilized on nanoparticles

Measured tensile strength of bio-healing specimens is presented in Fig. 9. The immobilized bacterial spores increased the tensile strength of the mixture by 49.5 & 33.7% as they were compared to the negative control sample and when they were compared to the positive control (with eggshells nanoparticles as a replacement for cement), respectively. In comparison to conventional concrete, bacteria-based self-healing concrete (particularly with ~ 10⁵ cells/mL *Bacillus subtilis*) increases split tensile strength by about 20–25% after 28 days [51 ].

MICP bacteria can enhance the cohesiveness of concrete through the process by producing calcium carbonate (CaCO_3_) as a product of their metabolic activity. This CaCO_3_ fills pores and microcracks in the concrete matrix and binds aggregate particles more tightly, resulting in increased bonding and strength [12–52]. The samples’ continuous rise in tensile strength suggests that microbial activity might improve the concrete matrix’s internal cohesiveness. It is known that biomineralization, especially the precipitation of calcium carbonate, fills holes and microcracks, which may explain the noted increases in tensile strength [53]. Concrete’s developing tensile strength could be explained by the creation of a dense interfacial transition zone (ITZ) between the natural aggregate and cement. The nanoparticles improve the matrix-aggregate bond because of their large specific surface area [54].

### Flexural strength

**Fig. 10:**
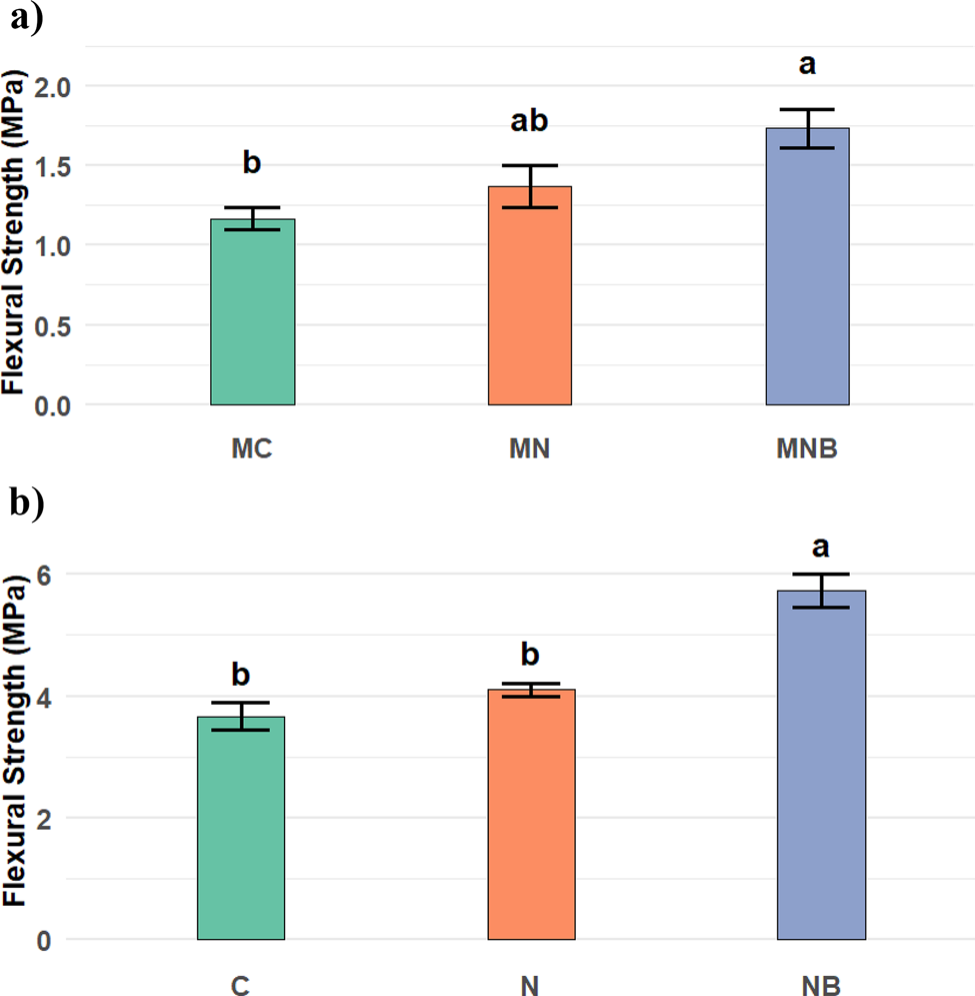
**a**) Shows the mean values of the flexural strength (MPa) of the mortar specimens after 28 days of water curing, expressed as Mean ± SE along with the corresponding 95% confidence intervals (CI). A one-way ANOVA revealed a statistically significant effect of the mixture type (F = 6.758, *p* = 0.029, ges = 0.693), indicating that the inclusion of nanoparticles and bacterial spores significantly enhanced the flexural strength of the mortar. MC: Refers to the mortar mixture without nanoparticles and without bacterial spores. MN: Refers to the mortar mixture with nanoparticles and without bacterial spores. MNB: Refers to the mortar mixture with bacterial spores immobilized on nanoparticles. **b**) Shows the mean values of the flexural strength (MPa) of the concrete specimens after 28 days of water curing, expressed as Mean ± SE along with the corresponding 95% confidence intervals (CI). Among the tested groups, NB specimens exhibited the highest flexural strength (5.73 ± 0. 27 MPa), followed by N (4.10 ± 0. 12 MPa), while C showed the lowest value (3.67 ± 0.23 MPa). A one-way ANOVA revealed a highly significant effect of the mixture type on flexural strength (F = 25.055, *p* = 0.001, ges = 0.893), indicating that the incorporation of nanoparticles and bacterial spores substantially enhanced the flexural strength of the concrete specimens. C: Refer to the concrete mixture without nanoparticles or bacterial spores. N: Refer to the concrete mixture with nanoparticles and without bacterial spores. NB: Refer to the concrete mixture with bacterial spores immobilized on nanoparticles

It was observed that immobilized bacterial spores increased the flexural strength of the mortar specimen mixture by 50 & 38.5% as they were compared to the negative and positive control samples, respectively, as presented in Fig. 10a. For the concrete specimen mixture, flexural strength increased by 47.5 & 36.9% as they were compared to the negative and positive control samples, respectively Fig. 10b. Our findings are supported by Digafe et al., who found that bacterial concrete outperformed conventional concrete by 35.15% in compressive strength, 24.32% in tensile strength, and 17.24% in average flexural strength [55].

The flexural strength results demonstrated that the EShN successfully enhanced the concrete tensile and flexural strength because of their surface characteristics, which raised the cohesiveness between the cement matrix and the filler surface [49]. The finer particle composition of EShN may be the cause of the higher flexural strength [56–59]. Using eggshells nanoparticles as a bacterial carrier is a promising material where it is considered a waste that improves the mechanical properties of produced composites, leading to better strength and stiffness mixtures and providing cheap raw material for the immobilization process.

The experimental results showed that the immobilized bacterial spores positively influenced the compressive, flexural, and tensile strengths of the concrete when they are compared to the negative and positive control samples.

Eggshells constitute a significant portion of food waste that is rich in calcium carbonate (CaCO_3_), which can replace a portion of cement or limestone in concrete. This lessens the requirement for extracting raw materials, such as limestone, from quarries. One of the biggest sources of CO_2_ emissions is the cement industry. Using eggshells powder in place of some of the cement lowers carbon emissions, helps mitigate climate change, and results in less expensive concrete products. It eliminates the need for expensive cement when used in the production of concrete [60]. Research indicates that adding eggshells powder to concrete can improve its durability and compressive strength while lowering long-term repair and maintenance costs [49]. The need for environmentally friendly building materials is rising. Eggshells waste added to concrete may appeal to markets for sustainable building [48]. Our technique is efficient to implement since it reduces the detrimental environmental effects of cement consumption by replacing cement or a portion of it with calcium carbonate-rich eggshell powder. Considering the bacteria we employ is spore-forming, easy to grow, and doesn’t require costly nutritional requirements for growth, we encourage its use in real-world situations, particularly in water structures or those situated alongside rivers and oceans. Immobilization of spores is a fast procedure that takes no more than thirty minutes and can be carried out on a large scale, for instance, inside barrels with a stirring device inside.

## Conclusions

Incorporating immobilized bacterial spores into the concrete matrix significantly enhanced its mechanical performance, increasing compressive, tensile, and flexural strengths by 45, 49.5, and 47.5%, respectively, compared with negative controls. These results highlight the potential of microbial-induced calcium carbonate precipitation (MICP) as a sustainable approach for developing durable and bio-healing concrete. Structural analyses using SEM, TEM, and XRD confirmed that eggshell nanoparticles (EShN) improved the microstructure due to their high surface area and nanoscale size, which facilitated uniform dispersion and promoted CaCO₃ formation within the matrix.

Moreover, utilizing eggshell waste as a nanoparticle source offers an ecofriendly and cost effective immobilization medium that protects bacterial spores from the harsh alkaline environment of concrete, ensuring sustained microbial activity. Overall, this study demonstrates that EShN based immobilization enhances mechanical properties and supports sustainable concrete production through microbial mineralization and waste reutilization. Bacterial concrete faces significant limitations in achieving long-term effectiveness practicality dry conditions. Additionally, the healing process only works for small cracks (under 0.5 mm) and takes weeks to months to complete, limiting its use in structural or time-sensitive applications. Ensuring adequate and uniform nutrient distribution is another obstacle, as nutrients degrade unevenly, while quality control remains problematic due to inconsistent bacterial activity.

## Data Availability

All data generated or analyzed during this study are included in this published article.
